# Lopinavir and Nelfinavir Induce the Accumulation of Crystalloid Lipid Inclusions within the Reservosomes of *Trypanosoma cruzi* and Inhibit Both Aspartyl-Type Peptidase and Cruzipain Activities Detected in These Crucial Organelles

**DOI:** 10.3390/tropicalmed6030120

**Published:** 2021-07-01

**Authors:** Leandro S. Sangenito, Miria G. Pereira, Thais Souto-Padron, Marta H. Branquinha, André L. S. Santos

**Affiliations:** 1 Laboratório de Estudos Avançados de Microrganismos Emergentes e Resistentes (LEAMER), Departamento de Microbiologia Geral, Instituto de Microbiologia Paulo de Góes, Universidade Federal do Rio de Janeiro (UFRJ), Rio de Janeiro 21941-901, Brazil; ibastefano@hotmail.com (L.S.S.); padron@gmail.com (T.S.-P.); 2 Laboratório de Ultraestrutra Celular Hertha Meyer, Instituto de Biofísica Carlos Chagas Filho, Universidade Federal do Rio de Janeiro (UFRJ), Rio de Janeiro 21941-901, Brazil; miriagpereira@gmail.com; 3 Instituto Nacional de Ciência e Tecnologia em Biologia Estrutural e Bioimagens, Rio de Janeiro 21941-901, Brazil; 4 Programa de Pós-Graduação em Bioquímica, Instituto de Química, Universidade Federal do Rio de Janeiro (UFRJ), Rio de Janeiro 21941-901, Brazil

**Keywords:** *Trypanosoma cruzi*, reservosomes, lipid inclusions, aspartyl-like peptidases, cruzipain, HIV-PIs

## Abstract

Several research groups have explored the repositioning of human immunodeficiency virus aspartyl peptidase inhibitors (HIV-PIs) on opportunistic infections caused by bacteria, fungi and protozoa. In *Trypanosoma cruzi*, HIV-PIs have a high impact on parasite viability, and one of the main alterations promoted by this treatment is the imbalance in the parasite’s lipid metabolism. However, the reasons behind this phenomenon are unknown. In the present work, we observed by transmission electron microscopy (TEM) that the treatment of *T. cruzi* epimastigotes with the HIV-PIs lopinavir and nelfinavir induced a huge accumulation of crystalloid-shaped lipids within the reservosomes, most of them deforming these key organelles. As previously reported, those structures are characteristic of lipid inclusions formed mostly of cholesterol and cholesterol-esters. The fractionation of nontreated epimastigotes generated two distinct fractions enriched in reservosomes: one mostly composed of lipid inclusion-containing reservosomes (Fraction B1) and one where lipid inclusions were much less abundant (Fraction B2). Interestingly, the extract of Fraction B2 presented enzymatic activity related to aspartyl-type peptidases 3.5 times higher than that found in the extract obtained from Fraction B1. The cleavage of cathepsin D substrate by this class of peptidases was strongly impaired by pepstatin A, a prototypical aspartyl PI, and the HIV-PIs lopinavir and nelfinavir. In addition, both HIV-PIs also inhibited (to a lesser extent) the cruzipain activity present in reservosomes. Finally, our work provides new evidence concerning the presence and supposed participation of aspartyl peptidases in *T. cruzi*, even as it adds new information about the mechanisms behind the alterations promoted by lopinavir and nelfinavir in the protozoan.

## 1. Introduction

Human immunodeficiency virus aspartyl peptidase inhibitors (HIV-PIs) have been the cornerstone of HIV-infected individuals’ treatment since its advent in the mid-1990s. In addition to impairing the replication and maturation of viral particles [1], the introduction of HIV-PIs to highly active antiretroviral therapy (HAART) also had an impact on opportunistic coinfections [2,3,4,5]. In this line, our research group studied the repositioning of HIV-PIs against *Trypanosoma cruzi*, where the most effective PIs, lopinavir and nelfinavir, had a huge effect on all life forms of the parasite (for a comprehensive reading, see the review [6]). One of the most common effects triggered by the HIV-PIs was alterations in the parasite’s lipid metabolism, where both lopinavir and nelfinavor induced a significant augment in the neutral lipid storage, as judged by incubation with Nile red fluorophore [6].

The exact mechanisms about how HIV-PIs can interfere with the lipid metabolism of *T. cruzi* remain unknown and deserve further study. Such a phenotype is very interesting to note, as disorders in lipid metabolism such as lipodystrophy and dyslipidemia are well-known complications reported in HIV-positive patients under prolonged HIV-PI regiment [7,8]. Similar alterations were also reported by our research group in *Leishmania amazonensis* promastigotes [9,10], reflecting a common metabolic alteration promoted by HIV-PIs in both lower (e.g., trypanosomatids) and higher eukaryotic organisms.

Regarding neutral lipids in *T. cruzi* epimastigotes, the major sterol produced is ergosterol. Remarkably, the parasite does not synthesize cholesterol, which needs to be acquired as well as all other macromolecules mainly through the cytostome–cytopharynx complex [11,12]. From both sites, cargo vesicles bud off and fuse with early endosomes [12]. After that, exogenous cholesterol is delivered to be stored in the reservosome, the last organelle of the *T. cruzi* endocytic pathway that accumulates a set of enzymes involved in lipid metabolism [13]. Indeed, the lipid inclusions in reservosomes are formed mostly of cholesterol and cholesteryl esters. Likewise, the stored cholesterol can be mobilized from the organelles in an opposite flow according to the parasite’s nutritional requirements [14,15,16]. Reservosomes are also the main site for the accumulation of a set of hydrolases, such as cruzipain, regarded as the main site of protein degradation [13]. Therefore, these lysosome-related organelles are crucial structures responsible for providing substrates to cellular metabolic demands and storing nutrients to be consumed during the metacyclogenesis process [12,13,17].

In the present work, we reported the side effects caused by treatment with the HIV-PIs lopinavir and nelfinavir on the storage of high amounts of lipids in the reservosomes of *T. cruzi* epimastigotes by transmission electron microscopy, as well as the inhibition of both aspartyl-type peptidase and cruzipain activities detected within these isolated key organelles.

## 2. Materials and Methods

### 2.1. Parasite Cultivation

*Trypanosoma cruzi* (Y strain) epimastigotes were cultivated for 3–4 days at 28 °C in brain heart infusion medium (BHI; Sigma-Aldrich Chemical Co., St. Louis, MO, USA) supplemented with 10% heat-inactivated fetal bovine serum (FBS; Gibco Life Technology, New York, NY, USA). Parasite growth was measured by direct cell counting in a Neubauer hemocytometer chamber. In all experiments, epimastigote forms were used in the exponential phase of growth.

### 2.2. Treatment of T. cruzi Epimastigotes with Aspartyl PIs

Viable epimastigotes (5 × 10^6^ cells/mL) were incubated at 28 °C for 72 h in the absence (control system) and in the presence of the concentration corresponding to the IC_50_ value of either lopinavir (3.8 µM) or nelfinavir (7.3 µM) [18]. The parasite viability was monitored by the motility and lack of Trypan blue staining.

### 2.3. Cell Fractionation

The cell fractions of nontreated epimastigotes were obtained according to Cunha-e-Silva and coworkers [19]. Briefly, epimastigotes in TMS buffer (20 mM Tris-HCl, pH 7.2, 2 mM MgCl_2_ and 250 mM sucrose) were disrupted by sonication on ice in an ultrasonic apparatus (Sigma, GEX 600 Model) using a standard probe. After differential centrifugation, the supernatant was mixed with an equal volume of 2.3 M sucrose in TMS buffer, deposited into a Beckman SW28 centrifuge tube, overlaid with 10 mL of 1.2 M, 10 mL of 1.0 M and 5 mL of 0.8 M sucrose (in TMS buffer) and centrifuged at 97,000× *g* for 150 min. The interfaces 0.8 M/1.0 M (B1) and 1.0 M/1.2 M (B2), the sample layer (B3) and the pellet (B4) were collected, diluted in TMS buffer and centrifuged at 120,000× *g* for 30 min. The pellets, named B1, B2, B3 and B4, were resuspended in TMS and stored at −20 °C until use.

### 2.4. Transmission Electron Microscopy (TEM) of Epimastigotes and Cell Fractions

Control parasites, lopinavir- and nelfinavir-treated parasites, and cellular fractions from the sucrose gradient were fixed in 2.5% glutaraldehyde in 0.1 M sodium cacodylate buffer, pH 7.2, for 1 h at room temperature, postfixed for 40 min in 1% OsO_4_ supplemented with 0.8% potassium ferrocyanide and 5 mM calcium chloride in 0.1 M cacodylate buffer. Samples were dehydrated in an acetone series and embedded in Epon. Ultrathin sections were stained with uranyl acetate and lead citrate and observed in a JEOL 1200 transmission electron microscope operating at 80 kV.

### 2.5. Aspartyl Peptidase Activity Assays in Reservosomes Fractions

The enzymatic activity over 7-methoxycoumarin-4-acetyl-Gly-Lys-Pro-IIe-Leu-Phe-Phe-Arg-Leu-Lys(DNP)-D-Arg amide substrate (cathepsin D substrate; Sigma-Aldrich Chemical Co., St. Louis, MO, USA) was determined using cell fraction extracts (B1–B4), which were obtained by repeated freeze-thawing cycles in a reaction buffer containing 0.2 M sodium phosphate, 0.1 M citric acid, 1 mM ethylenediamine tetraacetic acid (EDTA), 1% 3-[(3-cholamidopropyl)dimethylammonio]-1-propanesulfonate (CHAPS) and 10 μM L-*trans*-epoxysuccinyl-L-leucylamido-(4-guanidino)-butane (E-64), pH 4.0. Then, the fraction extracts were centrifuged at 10,000× *g* for 30 min at 4 °C, and the supernatants were immediately used to determine the protein content and the proteolytic activity. The protein concentration was determined by the method described by Lowry and coworkers [20], using bovine serum albumin (BSA) as standard. The cleavage of cathepsin D substrate was monitored continuously in a spectrofluorometer (SpectraMax Gemini XPS, Molecular Devices, CA, USA) using an excitation wavelength of 328 nm and an emission wavelength of 393 nm. A 200 μM stock solution of the cathepsin D substrate was prepared in DMSO. The reactions were started by the addition of the substrate (2 μM) to the extracts (40 μg protein) in reaction buffer in the presence and in the absence of pepstatin A (a prototypal aspartyl PI) at 10 μM. Alternatively, the extract corresponding to the reservosome fraction B2 was also submitted to the aspartyl PI diazo-acetyl-norleucinemethylester (DAN) and the HIV-PIs lopinavir and nelfinavir, all of them at 10 µM. The reaction mixtures were incubated at 37 °C for 1 h. The assays were controlled for self-liberation of the fluorophore over the same time interval [18].

### 2.6. Cruzipain Activity Assay in Reservosome B2 Fraction Extract

For this assay, the reservosome B2 fraction extract was obtained through repeated freeze-thawing cycles in a reaction buffer containing 70 µM of imidazole, 2 µM of dithiothreitol (DTT) and 1% CHAPS, pH 5.5. Then, the B2 extract was centrifuged at 10,000× *g* for 30 min at 4 °C, and the supernatants were immediately used to determine the protein content and the proteolytic activity. The hydrolysis of the fluorogenic substrate Z-Phe-Arg-amidomethylcoumarin (Sigma-Aldrich Chemical Co., St. Louis, MO, USA), commonly used to detect cathepsin B and L activities, including cathepsin-L-like cruzipain [21], was monitored continuously in a spectrofluorometer (SpectraMax Gemini XPS, Molecular Devices, CA, USA) using an excitation wavelength of 380 nm and an emission wavelength of 460 nm. The reaction was started by the addition of the fluorogenic substrate to the B2 fraction extract (10 μg protein) in the absence or in the presence (10 µM) of the selective and irreversible cysteine peptidase inhibitor E-64, lopinavir and nelfinavir. The reaction mixtures were incubated at 37 °C for 1 h and the assays controlled for self-liberation of the fluorophore over the same time interval.

### 2.7. Statistic and Graph Constructions

All experiments were performed in triplicate in three independent experimental sets. Data were analyzed statistically by means of one-way analysis of variance (ANOVA) using GraphPad Prism software 6.0 (GraphPad Software Inc., La Jolla, CA, USA). Graphs were made in the same program.

## 3. Results and Discussion

Previously, our research group observed the influence of lopinavir and nelfinavir on the accumulation and distribution of neutral lipids in *T. cruzi* epimastigotes. The visualization of lopinavir-/nelfinavir-treated parasites under fluorescence microscopy revealed the presence of many Nile red-stained lipid bodies, which were randomly distributed throughout the cytoplasm [22]. Subsequently, TEM analyses revealed the formation of lipid droplets in close contact with the endoplasmic reticulum of *T. cruzi* trypomastigotes treated with both lopinavir and nelfinavir. Indeed, Nile red staining confirmed the augment of the lipid content either grouped or, in some cases, randomly distributed throughout the cytoplasm of trypomastigotes [23]. Therefore, it is clear that treatment with HIV-PIs somehow affects the lipid metabolism of *T. cruzi*, as occurs in the treatment of HIV-positive patients [7,8]. However, the exact mechanism of how this phenomenon occurs in *T. cruzi* is completely unknown. Some possibilities of nonspecific toxic effects of HIV-PIs have been raised, but we can also presume that the aspartyl-type peptidases produced by the parasite can be a target and may be directly/indirectly involved in this lipid imbalance [6].

**Figure 1:**
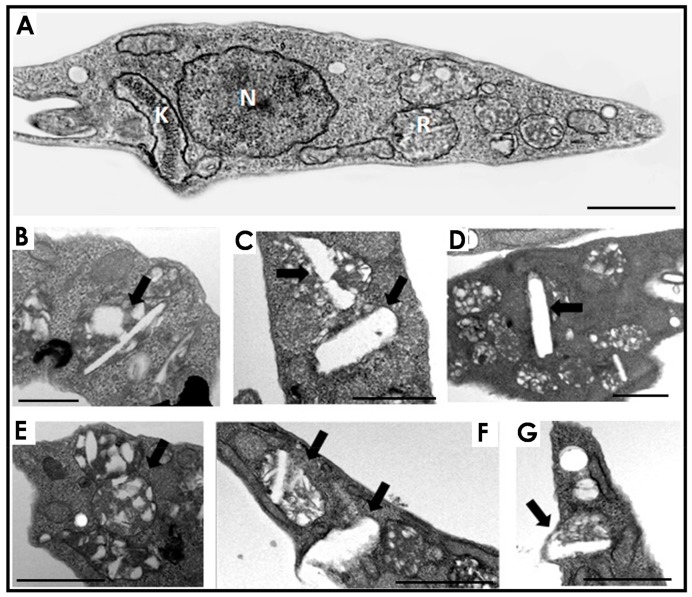
Effects of lopinavir and nelfinavir on the reservosomes of *T. cruzi* epimastigotes. (**A**) Transmission electron microscopy of control parasites revealed the normal morphology of organelles such as nucleus (N), kinetoplast (K) and reservosome (R). The treatment with lopinavir (**B**–**D**) and nelfinavir (**E**–**G**) induced a huge appearance of lipid inclusions inside the reservosomes (*black thick arrows*). Bars = 500 nm. The images are a representative set of three independent experiments.

With all these premises in mind, the main purpose of the present work was to verify the effects of HIV-PIs, more specifically lopinavir and nelfinavir, on the aspartyl-type activity present in the reservosomes isolated from epimastigote forms of *T. cruzi*. Reservosomes are the last organelles of the endocytic pathway in epimastigotes, being one of the sites for lipid storage [14]. Therefore, we firstly treated *T. cruzi* epimastigotes for 72 h with the IC_50_ doses of lopinavir and nelfinavir as previously determined for the Y strain [18] to analyze the effects of these PIs on the reservosome ultrastructure and lipid storage. Our TEM analyses revealed that lopinavir (Figure 1B–D) and nelfinavir (Figure 1E–G) induced changes in lipid traffic due to the unusual accumulation of lipids within the reservosomes. These exacerbated lipid inclusions were droplet-shaped (Figure 1B,E) or rod-shaped crystalloid bodies that deformed the organelles (Figure 1C,D,F,G).

The formation of these crystalloid lipid inclusions in *T. cruzi* reservosomes was observed by Sant’Anna and colleagues [24]. Later, the establishment of a direct correlation between serum concentration and the presence of these inclusions in the organelle was determined [14]. The progressive augment of serum concentration induced a proportional increase in lipid accumulation. On the opposite, lipid starvation was able to disassemble the reservosome crystalloid inclusions [15]. Starting from a reservosome-purified fraction, thin-layer chromatography and gas-chromatography mass-spectrometry analyses revealed that those lipid inclusions were composed mostly of cholesterol and cholesterol-esters. Thus, it is possible to assume in our work that the lipids accumulated in the reservosomes were composed mainly of cholesterol and cholesterol-esters. However, this accumulation was a result of the action of lopinavir/nelfinavir, and not the increase in the concentration of FBS, which remained at 10%. Similar to that found in *T. cruzi*, treatment of *L. amazonensis* promastigotes with nelfinavir [9] and lopinavir [10] also promotes alterations in lipid storage. Rebello and colleagues [10] observed a dose-dependent increase in the number of lipid inclusions in lopinavir-treated parasites, which was accompanied by a marked increase in cholesterol and cholesterol-ester content. Interestingly, those authors pointed out that lopinavir altered the sterol profile in *L. amazonensis* without inhibiting ergosterol biosynthesis, as this lipid content was not affected by the treatment.

**Figure 2:**
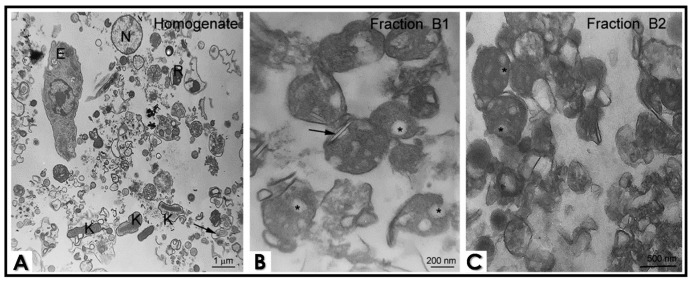
Ultrastructure of epimastigote cell fractionation by transmission electron microscopy. (**A**) Total homogenate shows cell debris, reservosomes (R), nuclei (N), whole epimastigote (E), membrane profiles, flagella (*black arrow*) and kinetoplast (K). Fractions B1 and B2 are enriched in reservosomes, although B1 presents more reservosomes containing lipid inclusions. (**B**) Cholesterol-rich inclusions are frequently observed in a flattened shape (*black arrows*) or as a rounded inclusion in the reservosome lumen (*asterisk*). (**C**) Reservosomes from Fraction B2 are more electron dense, and lipid inclusions are less abundant (*asterisk*), suggesting that there are subpopulations of these organelles. The images are a representative set of three independent experiments.

Considering that lipid storage in the reservosomes was somehow affected by lopinavir/nelfinavir treatment, we asked whether: (1) there would be aspartyl-type peptidases within the reservosomes; (2) there would be differences in the enzyme activity in reservosome populations rich and poor in lipid content; (3) the detected activity was inhibited by classic aspartyl PIs and HIV-PIs. To answer these questions, we first fractionated the cellular content of nontreated epimastigotes (Figure 2A) in a sucrose gradient with TMS buffer so that we obtained four cellular fractions, designated as B1 to B4. Fraction B1 was mostly composed of lipid-rich reservosomes. Cholesterol-rich inclusions were frequently observed in a flattened shape (black arrows) or as a rounded inclusion in the reservosome lumen (asterisk) (Figure 2B). Reservosomes of Fraction B2 were more electron dense and less abundant in neutral lipid content (asterisk) (Figure 2C). The morphological integrity of both subpopulations of these organelles remained stable after the cell fractionation process. B3 contained microsomal fractions and glycosomes, while B4 was formed by acidocalcisomes, mitochondria profiles and flagellum and membrane debris (data not shown).

**Figure 3:**
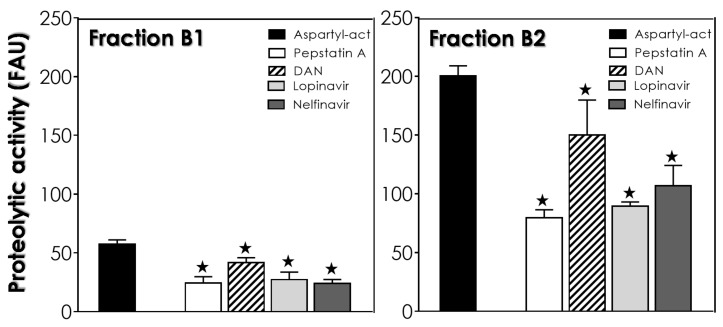
Effects of lopinavir and nelfinavir on the aspartyl-type activity of Fractions B1 and B2 of the reservosomes. The aspartyl-type peptidase activity (act) was quantified fluorometrically over cathepsin D substrate in the absence (*control: black bars*) or in the presence of lopinavir and nelfinavir at 10 μM. The classical aspartyl peptidase inhibitors pepstatin A and DAN at 10 µM were also included in the assays. The results are expressed as fluorescence arbitrary units (FAUs), which represent the fluorescence liberated by the fluorophore in 60 min of reaction. Values represent the mean ± standard deviation of three independent experiments. The black stars represent significant statistical differences in systems with pepstatin A compared to the respective controls (*p* < 0.01).

All fractions of nontreated epimastigotes were tested for aspartyl-type activity using cathepsin D substrate. Fraction B3 presented high detected activity with the classic aspartyl PI, pepstatin A, reducing the proteolytic activity by 40.6%, while B4 showed almost no detectable activity (data not shown). The extract of the reservosome fraction (B1) presented low aspartyl-type peptidase activity, with pepstatin A impairing the proteolysis by 57.7% (Figure 3). Conversely, the extract of Fraction B2 showed high detected enzymatic activity (3.5 times higher than that found for B1) with pepstatin A reducing the proteolysis by 60.3% (Figure 3). Likewise, the HIV-PIs lopinavir and nelfinavir also impaired the aspartyl-type peptidase activity present in the extract from Fraction B2. It is interesting to note that the inhibition reached by lopinavir (55.4%) and nelfinavir (46.7%) was much more drastic than that found for DAN (26.6%), another classical aspartyl PI routinely used in enzymatic assays (Figure 3). Similarly, the low activity in Fraction B1 was also impaired by DAN (27.3%), lopinavir (52.7%) and nelfinavir (58.1%) (Figure 3). These data indicate an inverse correlation, where aspartyl-type peptidases are preferentially present/active in reservosomes that have little lipid content (B2 fraction) and that those enzymes are targets for HIV-PIs.

Looking into the genome of *T. cruzi*, three different genes for aspartyl-type peptidases were reported [25,26]. In 2018, our group modeled the three-dimensional structure of an aspartyl peptidase of *T. cruzi* and performed docking binding experiments with the HIV-PIs. The docking assays revealed that HIV-PIs bind to the active site of the enzyme, with lopinavir and ritonavir having greater affinity [27]. Indeed, several works have demonstrated the potential inhibition of HIV-PIs on aspartyl-type peptidases produced by both epimastigotes and trypomastigotes of *T. cruzi*; however, these enzymatic assays were conducted with the crude extract of the parasites, without distinguishing where these enzymes could be localized (reviewed in [6]). Here, we found an enzymatic activity reported to aspartyl-type peptidases mainly in the lipid-poor reservosomes (Fraction B2) of *T. cruzi*, where this activity was strongly inhibited by lopinavir and nelfinavir. The proteomic of *T. cruzi* reservosomes launched in 2009 [13] revealed 709 proteins, 456 of these presenting predicted functions. None of them were identified as a classical aspartyl peptidase; however, it may be among the 253 remaining initially classified as hypothetical proteins. In this context, we must take into account the methodological limitations of the study and almost nonexistent knowledge about aspartyl peptidases in *T. cruzi* at the time (in 2009). Other issues to take into account are that the authors used the B1 fraction to perform the proteomics when there is a lot more aspartyl-type activity in the B2 fraction and the difficulty in knowing whether the proteins identified in the reservosomes are native to them or whether they are transient proteins.

Recently, a presenilin-like transmembrane aspartyl enzyme was reported in *T. cruzi*. The presenilin-like peptidase was detected in the flagellar pocket, endoplasmic reticulum and reservosomes [28]. Its subcellular localization suggests participation in secretion and/or endocytic trafficking. Interestingly, serum deprivation leads to the upregulation of *T. cruzi* presenilin-like protein expression but is mostly concentrated in the regions of the flagellum and the flagellar pocket region near the kinetoplast [28]. The discovery of an aspartyl peptidase by the authors in the endoplasmic reticulum and in reservosomes strengthens our previous and current findings concerning the inhibition of aspartyl peptidases by HIV-PIs and lipid storage imbalance in those organelles. Indeed, TEM analyses revealed that treatment with lopinavir/nelfinavir led to lipid accumulation in the endoplasmic reticulum of *T. cruzi* trypomastigotes [23]. Concerning the presence of presenilin-like peptidase in the reservosomes, we cannot exclude the possibility of two additional aspartyl peptidases predicted on the *T. cruzi* genome: a membrane signal peptide peptidase (SSP) and Ddi-1 aspartyl peptidase [25]. For this last one, it was previously demonstrated that it is a target for HIV-PIs in *L. major* [29].

**Figure 4:**
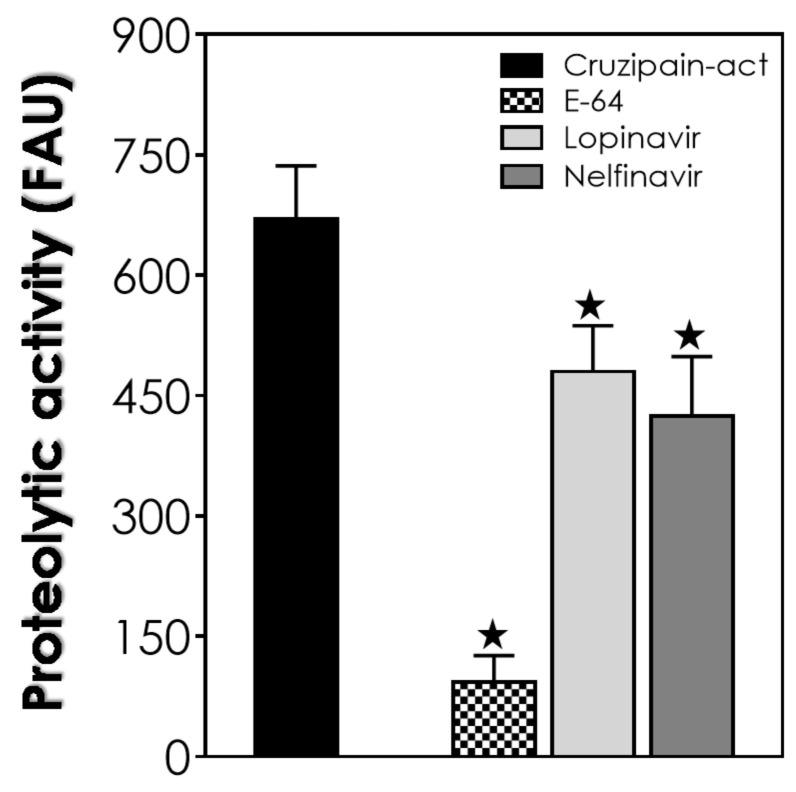
Effects of lopinavir and nelfinavir on cruzipain activity present on the Fraction B2 of the reservosomes of *T. cruzi*. The cruzipain activity (act) was quantified fluorometrically over a specific substrate used to detect cathepsin-L-like cruzipain activity in the absence (*control: black bars*) or in the presence of 10 µM of lopinavir, nelfinavir and the classical cysteine peptidase inhibitor E-64. The results are expressed as fluorescence arbitrary units (FAUs), which represent the fluorescence liberated by the fluorophore in 60 min of reaction. Values represent the mean ± standard deviation of three independent experiments. The black stars represent significant statistical differences in systems with pepstatin A compared to the respective controls (*p* < 0.01).

Once the aspartyl-type activity present in Fraction B2 was impaired by HIV-PIs, we wondered if these compounds would be able to inhibit the cruzipain within these organelles. Cruzipain, a cysteine peptidase, is the major enzyme of *T. cruzi*, found in large quantities inside the epimastigote reservosomes. This cysteine peptidase is involved in the interaction process with host cells, escape from the immune system, differentiation processes and nutritional protein degradation [30]. As expected, a high activity was detected in the B2 fraction extracts when using a specific substrate for the cathepsin-L-like cruzipain (Figure 4). The classical cysteine peptidase inhibitor E-64 drastically reduced (around 86%) the cruzipain activity detected in the B2 extract. On a smaller scale, nelfinavir impaired the cruzipain substrate degradation by 36.7%, while lopinavir reduced the proteolysis by only 28.4% (Figure 4).

In the study of Bellera and coworkers [31], the authors applied the computation of molecular topological descriptors and linear discriminant analysis for the search of novel compounds against *T. cruzi*, focusing on cruzipain as the main parasite target. Among the compounds preselected, the HIV-PI saquinavir was one of them, where docking studies and simulations predicted interactions with cruzipain. Corroborating these findings, the in vitro activity of the partially purified cruzipain was abolished by saquinavir at 100 μM [30]. In this same line of thinking, nelfinavir at 10 μM reduced the cruzipain-like activity present in *T. cruzi* trypomastigotes total extracts by approximately 32% [6].

## 4. Conclusions

In the present study, we reported that lopinavir and nelfinavir induce changes in lipid traffic and probably in epimastigote metabolism by the accumulation of crystalloid lipid inclusions in reservosomes, as observed in ultrathin sections. The similarity of these electron-lucent structures to previous analyses of reservosomes suggests that they are also constituted by cholesterol and cholesteryl-ester lipids [14,24]. After that, we decided to concentrate on studies with the reservosomes of untreated parasites. Interestingly, extracts from isolated reservosomes rich in lipid content (Fraction B1) showed low activity related to aspartyl-type peptidases. Conversely, the lipid-poor fraction (B2) showed high proteolytic activity, with great inhibition by pepstatin A and lopinavir/nelfinavir. This inverse correlation led us to suppose that the aspartyl-type peptidases present in the reservosomes could somehow be (directly and/or indirectly) linked to the processes of cholesterol mobilization by the endocytic pathway in the protozoan, where the inhibition of the enzymes by HIV-PIs led to lipid accumulation in the organelles, as evidenced by TEM analyses. This assumption is reinforced by the recent work of Lechuga and coworkers [28], who suggested the participation of an aspartyl-like peptidase in secretion and/or endocytic trafficking due to its localization on the flagellar pocket, endoplasmic reticulum and reservosomes. However, we cannot exclude the possibility of other targets for HIV-PIs or even nonspecific toxic effects. Indeed, lopinavir and nelfinavir also affected the cruzipain activity within B2 reservosomes. So, these HIV-PIs are able to provoke an imbalance in both proteolysis and lipid storage. Therefore, further studies are needed to better elucidate the effects of HIV-PIs. Finally, the work presents new information about the participation of aspartyl peptidases in *T. cruzi,* as well as new evidence of the mechanism of action of HIV-PIs, specifically lopinavir and nelfinavir, in the parasite.

## Data Availability

Not applicable.
